# Recombinant human proinsulin expression in *Pichiapastoris *using PGK promoter

**DOI:** 10.1186/1753-6561-8-S4-P88

**Published:** 2014-10-01

**Authors:** Enedina Nogueira de Assunçao, Santos Maria Cristina dos, Torres Fernando Araripe, Astolfi-Filho Spartaco

**Affiliations:** 1 Divisão de Biotecnologia, Centro de Apoio Multidisciplinar Universidade Federal do Amazonas, UFAM Manaus, AM, Brazil; 2 Instituto de Ciências Biológicas, Universidade Federal do Amazonas, UFAM Manaus, AM, Brazil; 3 Departamento de Biologia Celular, Universidade de Brasíli,a Brasília, DF, Brazil

## Background

There are indications, according to WHO, that diabetes will reach 333 million people in 2025, and already is among one of the main death causes in the world. In 2030 in Latin America, diabetes mellitus will be the second cause of deaths [1]. The actions to increase the production of insulin are strategic to respond the high demands for what we have to face in the near future [2]. *P. pastoris *methylotrophic yeast is being very used in the last decades to express heterologous genes to biotech uses [3]. The main expression vectors to *Pichia pastoris *are based in the strong promoter from *AOX1 *gene which codifies alcohol oxidize enzyme. However there is a search for new promoters than *AOX1 *due to certain disadvantages as the use of methanol as inducer that is toxic and originates undesirable subproducts during its metabolism [4]. This work has the main objective in to express in high levels the human proinsulin in *Pichia pastoris *using PGK promoter.

## Methods

The human proinsulin codifing sequence was chemically synthesized (Genone inc.) with *P. pastoris *optimized codons. The synthesized sequence was subcloned under PGK promoter control in expression vector together with the codifying region from *Saccaromyces cerevisiae *factor-α signal peptide in order to have proinsulin expression/secretion. After linearization the vector containing the expression cassete was introduced in *P. pastoris *GS115 by electroporation. To select clones, with multicopies of expression cassette, was realized a zeocin resistance test in plate [5]. Resistant clones to 100 μg/mL of zeocin were selected and submitted to crescent concentrations of the antibiotic: 500, 1000 and 2000 μg/mL. The recombinant clones that produced/secreted more proinsulin was detected by imunodot procedure using antibody anti-insulin/proinsulin (Pierce-D3E7/5B6/6). Selected recombinant clones were cultivated in YPD medium for 24 hours in 30°C at 200rpm and the supernatants were clarified by centrifugation and analyzed by ELISA and Western-blotting.

## Results and conclusion

From 200 zeocin (100 µg/mL) resistant *P. pastoris *clones 25 were able to grow in 2000 µg/mL which indicates that they have integrated several expression/ secretion cassetes in their genomes. From those one was notable for its higher human proinsulin expression/secretion in both experiments (imunodot and ELISA). The proinsulin overexpression of this selected clone was confirmed by PAGE and Western-blotting with anti-insulin/anti-proinsulin antibody. Additional experiments are in process in order to standardize the fermentative process for expression and secretion of human proinsulin as well as confirm recombinant protein amino acid sequence.
